# Inhibition of Carrageenan-Induced Acute Inflammation in Mice by Oral Administration of Anthocyanin Mixture from Wild Mulberry and Cyanidin-3-Glucoside

**DOI:** 10.1155/2013/146716

**Published:** 2013-01-17

**Authors:** Neuza Mariko Aymoto Hassimotto, Vanessa Moreira, Neide Galvão do Nascimento, Pollyana Cristina Maggio de Castro Souto, Catarina Teixeira, Franco Maria Lajolo

**Affiliations:** ^1^Laboratório de Química e Bioquímica e Biologia Molecular de Alimentos, Departamento de Alimentos e Nutrição Experimental, FCF, Universidade de São Paulo, Avenida Professor Lineu Prestes 580, Bloco 14, 05508-000 São Paulo, SP, Brazil; ^2^Núcleo de Apoio à Pesquisa em Alimentos e Nutrição (NAPAN), Universidade de São Paulo, 05508-000 São Paulo, SP, Brazil; ^3^Laboratório de Farmacologia, Unidade de Inflamação, Instituto Butantan, Avenida Vital Brasil 1500, 05503-000 São Paulo, SP, Brazil

## Abstract

Anthocyanins are flavonoids which demonstrated biological activities in *in vivo* and *in vitro* models. Here in the anti-inflammatory properties of an anthocyanin-enriched fraction (AF) extracted from wild mulberry and the cyanidin-3-glucoside (C3G), the most abundant anthocyanin in diet, were studied in two acute inflammation experimental models, in the peritonitis and in the paw oedema assays, both of which were induced by carrageenan (cg) in mice. In each trial, AF and C3G (4 mg/100 g/animal) were orally administered in two distinct protocols: 30 min before and 1 h after cg stimulus. The administration of both AF and C3G suppresses the paw oedema in both administration times (*P* < 0.05). In the peritonitis, AF and C3G reduced the polymorphonuclear leukocytes (PMN) influx in the peritoneal exudates when administered 1 h after cg injection. AF was more efficient reducing the PMN when administered 30 min before cg. Both AF and C3G were found to suppress mRNA as well as protein levels of COX-2 upregulated by cg in both protocols, but the inhibitory effect on PGE_2_ production in the peritoneal exudates was observed when administered 30 min before cg (*P* < 0.05). Our findings suggest that AF and C3G minimize acute inflammation and they present positive contributions as dietary supplements.

## 1. Introduction

Anthocyanins, glycosylated polyhydroxy and polymethoxy derivates of flavilium salt are natural colorants belonging to the flavonoid family and largely intaken from vegetable foods [1]. These pigments are responsible for the pink, red, violet, and blue colours in the flowers, fruits, and vegetables. There is a great variety of anthocyanins spread in the nature but only six are the most common: cyanidin, pelargonidin, malvidin, peonidin, petunidin, and delfinidin [2]. Interest in biological effects of anthocyanins has increased during the last decade because of increasing evidence demonstrating their potential therapeutic effects. Some anthocyanins have demonstrated to inhibit the growth of cancerous cells [3–5], to decrease hyperglycemic levels [6] and to promote antiobesity effects [7, 8]. Furthermore, anthocyanins possess antioxidant [9, 10] and anti-inflammatory [11–13] properties. This group of compounds has been demonstrated to modulate inflammation process dependent on the COX-2 pathway *in vitro* experimental protocols [14–18] and through the inhibition of nitric oxide biosynthesis [10].

Wild black mulberry (*Morus nigra* L.) extracts contains high level of anthocyanins. The identified anthocyanins are mainly cyanidin-3-glucoside (C3G), and in minor level cyanidin-3-rutinoside and pelargonidin derivate [19]. We previously reported that the anthocyanin-enriched extract (AF) obtained from wild black mulberry increased the plasma antioxidant capacity and the plasma catalase activity after oral intake in human [20]. Also, AF demonstrated inhibitory effect on the migration and invasion of a human lung cancer cell [5]. However, there are few studies that use *in vivo* experimental protocols, in order to demonstrate if oral intake of anthocyanins could affect inflammation. Since the anthocyanin is commonly intake daily from vegetable foods, it is important to establish evidence for the effect of anthocyanin consumption on health.

Inflammatory responses are a series of well-coordinated events that depend on the increase in vascular permeability and sequential release of inflammatory mediators, leading to oedema and arrival of inflammatory leukocytes to the site of inflammation, where neutrophils and macrophages are known to recruit and play pivotal roles in acute and chronic inflammation, respectively [21]. Cyclooxygenases (COXs) are the key enzymes in the synthesis of lipid mediators called prostaglandins observed in inflammation events. COXs convert free arachidonic acid, following its release from membrane phospholipids by phospholipases A_2_, to prostaglandin H_2_, the common precursor for all prostanoids. Nowadays, there are three COX isoforms named COX-1, COX-2, and COX-3 [22, 23]. COX-1 is a housekeeping enzyme, constitutively expressed in most mammalian tissues, and it is responsible for maintaining normal cellular physiologic functions. COX-2 is also present at a basal level in certain tissues, but its expression is induced in inflammatory cells and tissues in response to cellular activation by endotoxin, cytokines, mitogens, and other stimulus [24, 25]. COX-2 is the main enzyme providing a mechanism for the generation of proinflammatory prostanoids, such as prostaglandin E_2_ (PGE_2_), a potent vasodilator, which enhances oedema formation [26, 27]. COX-3, in turn, has been cloned [28, 29], but its function have yet to be well studied.

Therefore, in this study, we have examined, in mice, the anti-inflammatory activity of oral administration of an anthocyanin-enriched extract obtained from mulberry and its major component, the C3G, in the acute inflammation, peritonitis and paw oedema assays, induced by carrageenan, mainly on COX-2 mRNA and protein expression and PGE_2_ production.

## 2. Material and Methods

### 2.1. Mulberry Anthocyanin Preparations

The anthocyanin-enriched fraction (AF) was prepared from wild black mulberry according to the previously published method [19]. Briefly, the sample (approximately 5 g) was extracted three times with 100 mL of methanol:water:acetic acid (70 : 30 : 5, v : v : v) (Brinkmann homogeniser, Polytron-Kinematica GmbH, Kriens-Luzern, Sweden) in an ice bath. The homogenate was filtered under reduced pressure through filter paper (Whatman number 06). The methanol extract obtained was concentrated, under vacuum until methanol content elimination, using a rotary evaporator (Rotavapor RE 120, Buchi, Flawil, Sweden) and made up to 50 mL with distilled water. The extract (25 mL) was passed through polyamide (CC-6, Macherey-Nagel, Germany) column (10 g/60 mL) previously conditioned with 50 mL of methanol and 100 mL of distilled water. Impurities were washed out with distilled water and retained flavonoids were eluted with 120 mL of methanol acidified with 0.1% HCl. The flow rate through the columns was controlled by means of a vacuum manifold Visiprep 24DL (Supelco, Bellefonte, PA). The eluate was evaporated to dryness under reduced pressure at 40°C and dissolved in distilled water prior administration. This fraction corresponds to AF. C3G was further purified from AF according to Chen et al. [5] by passing it through a Bio-Gel P-2 column (40 cm × 2.5 cm) (Bio-Rad Laboratories, Hercules, CG), eluting it with aqueous acetic acid, pH 2.5, and monitoring it by spectrophotometer at 520 nm (Hitachi L-4000 UV-vis detector). The fraction corresponding to C3G, which was confirmed by HPLC-DAD, was collected and lyophilized. C3G was dissolved in distilled water prior to administration. 

### 2.2. Anthocyanin Quantification

**Figure 1:**
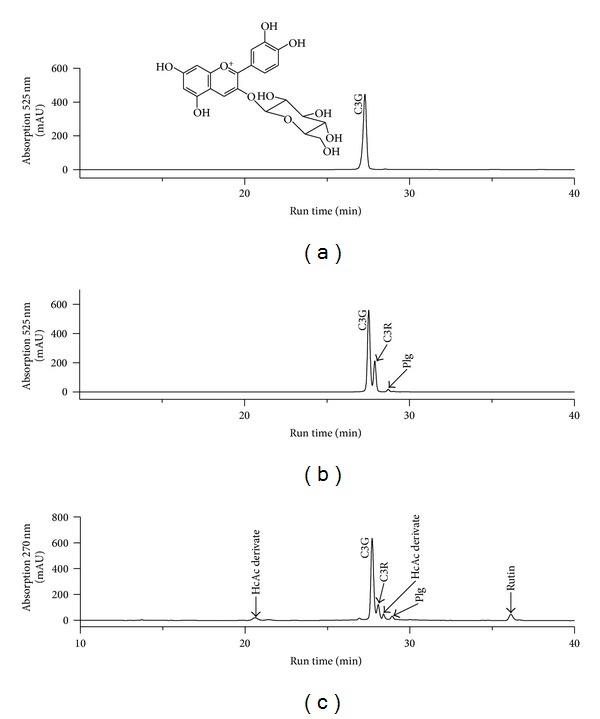
HPLC-DAD of cyanidin-3-glucoside (C3G) at 525 nm (a) and anthocyanin profile of AF at 525 nm (b) and 270 nm (c). Peaks were identified by MS/MS as C3G (structure showed), cyanidin-3-rutinoside (C3R), and rutin. Abbreviations: Hydroxycinnamic acid derivate (HcAc derivate) and pelargonidin (Plg).

For anthocyanin quantification, aliquots of AF and C3G were diluted with methanol: acetic acid (99 : 5, v : v) and filtered through a 0.45 *μ*m PTFE filter (Milipore Ltd., Bedford MA) prior to quantification by HPLC-DAD [19]. The column used was a Prodigy 5 *μ*m ODS3 (250 mm × 4.6 mm i.d., Phenomenex Ltd.) and elution solvents were (A) water : THF : TFA (98 : 2 : 0.1, v : v : v) and (B) acetonitrile. Solvent gradient consisted of 8% B at the beginning, 10% at 5 min, 17% at 10 min, 25% at 15 min, 50% at 25 min, 90% at 30 min, 50% at 32 min, and 8% at 35 min (run time, 35 min). Eluates were monitored at 270 and 525 nm. Flow rate was 1 mL/min; column temperature was 30°C. Peak identification was performed by comparison of retention times and diode array spectral characteristics with the standards and the library spectra. Cochromatography was used when necessary. C3G, C3R, and pelargonidin (Plg) (Extrasynthese, Genay, France) were used as standard. The total anthocyanin content of AF was expressed as C3G equivalent. The anthocyanin composition of AF is 85% C3G, 12% C3R, and 3% Plg derivate and they were previously identified by LC-MS [19]. The anthocyanin profile of AF and the purity of C3G are shown in Figure 1.

### 2.3. Animals

Male Swiss mice, weighing 18–20 g (approximately four weeks old), were acclimated to housing for at least 1 week prior to investigation. The night before the experiment, food was withdrawn from the cages but water was given *ad libitum*. Animals were randomly assigned to each treatment group and all testing was performed between 8:00 and 9:00 a.m. All animals were handled and experiments were conducted in accordance to the Guidelines for Animal Experimentation of the University of São Paulo, Brazil, after approval by the Ethics Committee of the Pharmacy Faculty of the University of São Paulo (Protocol number 53, FCF-USP).

### 2.4. Carrageenan-Induced Paw Oedema in Mice

To assess the effects of the AF and C3G on acute inflammation, the animals were deprived of food overnight and orally administered with an aqueous solution using an intragastric tube as described below.

AF Group: 200 *μ*L of the AF (4 mg C3G equiv/100 g body weight) were administered 30 min before (*n* = 8) and 1 h after (*n* = 8) intraplantar (i.pl.) injection of 50 *μ*L cg in saline (0.5% m/v) into the left hind paw. 

C3G Group: 200 *μ*L of C3G (4 mg/100 g body weight) were administered 30 min before (*n* = 8) and 1 h after (*n* = 8) i.pl. injection of 50 *μ*L carrageenan (cg) in saline (0.5% m/v) into the left hind paw. 

Control Group: 200 *μ*L of saline were administered 30 min before (*n* = 8) and 1 h after (*n* = 8) i.pl. injection of 50 *μ*L cg in saline (0.5% m/v) into the left hind paw.

Indomethacin Group: indomethacin (1 mg/kg, i.v) was administered 30 min before (*n* = 8) and 1 h after (*n* = 8) injection of 50 *μ*L cg in saline (0.5% m/v) into the left hind paw.

The contralateral paw was injected with 50 *μ*L of saline solution and used as a control. The volumes of both hind paws were measured by plethysmometry (model 7140 plethysmometer, Ugo Basile, Italy) 1, 2, 3, 4, and 5 h after the injection of cg. The results were reported as the percent inhibition of the volume increase to be compared with the preinjection paw volume. Mean values of treated groups were compared with mean values of a control group and analyzed using statistical methods.

### 2.5. Carrageenan-Induced Peritonitis in Mice

The animals were deprived of food overnight and orally administered with one of the following solutions.

AF Group: 200 *μ*L of the AF (4 mg C3G equiv/100 g body weight) were administered 30 min before (*n* = 8) and 1 h after (*n* = 8) intraperitoneal (i.p.) injection of 1 mL of cg in sterile saline (0.3%, m/v). 

C3G Group: 200 *μ*L of the C3G (4 mg/100 g body weight) were administered 30 min before (*n* = 8) and 1 h after (*n* = 8) i.p. injection of 1 mL of cg in sterile saline (0.3%, m/v). 

Carrageenan Control Group: 200 *μ*L of saline solution were administered 30 min before (*n* = 8) and 1 h after (*n* = 8) i.p. injection of 1 mL of cg in sterile saline (0.3%, m/v).

Indomethacin Group: 200 *μ*L indomethacin (4 mg/100 g body weight) were administered 30 min before (*n* = 8) and 1 h after (*n* = 8) i.p. injection of 1 mL of cg in sterile saline (0.3%, m/v).

Saline Control Group: 200 *μ*L saline solution were administered 30 min before (*n* = 8) and 1 h after (*n* = 8) i.p. injection of 1 mL of sterile saline solution.

Three hours after cg injections, the animals were killed by overexposure to CO_2_ and the peritoneal exudate was withdrawn after washing the peritoneal cavity with 2 mL of saline solution. Aliquots of the washes were used to determine total cell counts. An aliquot of the 1 × 10^6^ and 3 × 10^6^ cells was centrifuged at 800 *g*/6 min/22°C and used for COX-2 expression analysis by western blotting and RT-PCR, respectively. The supernatant was used for PGE_2_ quantification.

### 2.6. Leukocyte Harvesting and Counting

Aliquots of the peritoneal washes were used to determine total cell counts in a Newbauer chamber after dilution (1 : 20, v : v) in Turk's solution (0.2% crystal violet dye in 30% acetic acid). For differential cell counts, cytospin preparations were stained with Hema^3^ stain. Differential cell counts were performed by counting at least 100 cells, which were classified as either polymorphonuclear or mononuclear cells, based on conventional morphological criteria.

### 2.7. Western Blotting

The precipitate of cells (1 × 10^6^) was lisate with 100 *μ*L of sample buffer [30] and heated for 10 min/100°C. An aliquot of 14 *μ*L of the lisate was separated on SDS-polyacrylamide gels (10%) at 150 V and electrophoretically transferred to nitrocellulose membrane (GE Healthcare, Buckinghamshire, UK). The membrane was blocked with 5% nonfat milk in Tris buffered saline with 0.05% Tween 20 and incubated 1 h at room temperature with the antibody against COX-2 (1 : 1500) (Cayman Chemicals, Ann Arbor, MI) followed by incubation in the same buffer with the appropriate anti-rabbit horseradish peroxidase-conjugated secondary antibody (GE Healthcare, Buckinghamshire, UK) for 1 h at room temperature (1 : 1500). Further, the membrane was also incubated with the antibody against *β*-actin (1 : 2000) (Sigma, St. Louis, USA) followed by incubation with the anti-mouse secondary horseradish peroxidase-conjugate (1 : 2000) (GE Healthcare, Buckinghamshire, UK). Immunoreactive bands were detected using ECL kit (GE Healthcare, Buckinghamshire, UK). Densities of the bands were determined by a GS 700 Densitometer (Bio-Rad Laboratories, Richmond, CG) using the image analysis software from Molecular Analyst (Bio-Rad Laboratories, Richmond, CG).

### 2.8. RNA Preparation and Reverse Transcription-Polymerase Chain Reaction (RT-PCR)

Cells (3 × 10^6^) were washed once with sterile saline and mixed with 500 *μ*L of Trizol reagent (Invitrogen, Rockville, MD, EUA) and the RNA was extracted according to the manufacturer's instructions. Complementary DNA was synthesized using an Improm-II Reverse Transcription System (Promega, Madison, WI, USA) according to the manufacturer's instructions and conducted at a thermocycler Gene AMp (PCR System 2400, Applied Biosystems). PCR was performed by denaturing at 94°C for 60 s, annealing at 57°C (COX-2) and 60°C (*β*-actin) for 1 min and by extension at 72°C for 60 s. Thirty additional cycles for COX-2 and 25 cycles for *β*-actin were used for amplification. The primer pairs used for analysis were 5′-TTTGTTGAGTCGTTCGCCGGACGGA-3′ and 5′-CGGTATTGAGGAGAAGAGATGGGATT-3′ for sense and antisense primers of the COX-2 gene, respectively [31]; 5′-TGGAATCCTGTGGCGTCCGTGAAAC-3′ and 5′-TAAAACGCGGCTCGGTAACGGTCCG-3′ for sense and antisense primers of the *β*-actin gene, respectively [32], used as an inner control. 

### 2.9. PGE_2_ Quantification

Concentrations of PGE_2_ were determined by a specific enzyme immunoassay [33] using a commercial kit (Cayman Chemical Company, Ann Arbor, MI). The extraction of PGE_2_ was performed on Sep Pak C18 columns (Waters Corporation, Milford, MA) and eluted with ethanol. In brief, 50 *μ*L aliquots of each extracted sample were incubated with the PGE_2_ conjugated with acetylcholinesterase and the specific rabbit antiserum in 96-well plates, coated with anti-rabbit IgG mouse monoclonal antibody. After addition of the substrate, the absorbance of the samples was recorded at 405 nm in a microplate reader (Labsystem Multiscan), and concentrations of eicosanoids were estimated from standard curves.

### 2.10. Statistical Analysis

Results were presented as mean ± EPM. The statistical analyses were performed by one way analysis of variance (ANOVA) and *Tukey posthoc test* for comparison, using the Statistic software package version 5.0 (StatSoft, Inc.). Results were considered statistically significant for *P* values <0.05.

## 3. Results and Discussion

### 3.1. Effect of C3G and AF on Carrageenan-Induced Paw Oedema

The oral dose of both extracts and the two protocols applied in this study (30 min before or 1 h after inflammation stimulus) were chosen in order to provide high concentration of C3G in the plasma based in its rapid absorption and excretion [20].

**Figure 2:**
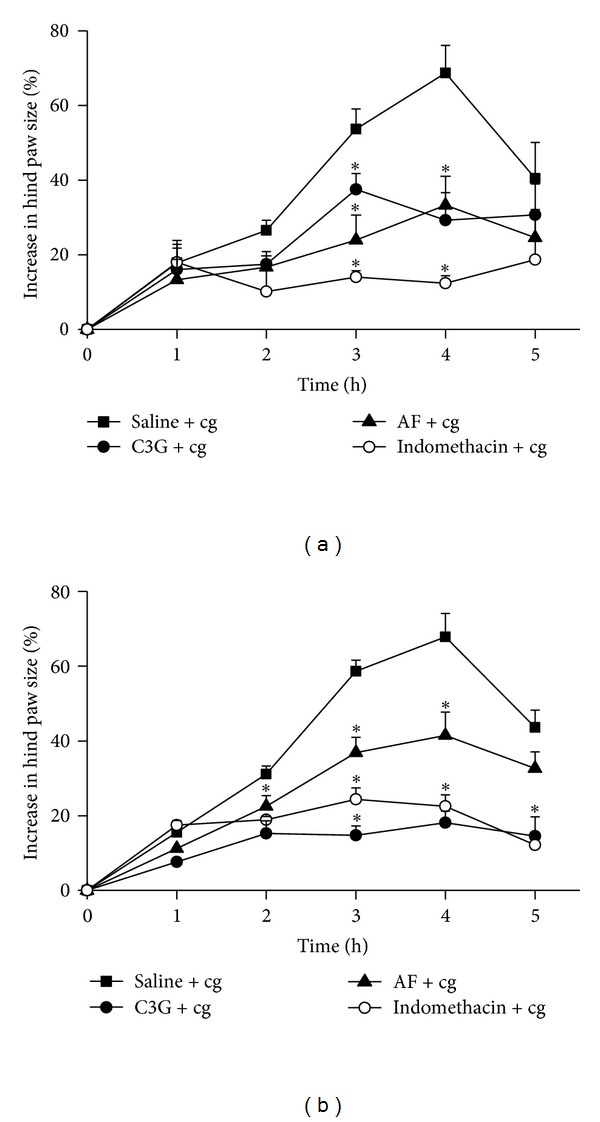
Effect of C3G and AF on carrageenan-induced paw oedema. Footpad oedema was induced by injection of cg (0.5% w/v in saline, i.pl.) and was evaluated by plethysmometry. C3G or AF (4 mg/100 g body weight) or indomethacin (1 mg/kg, i.v.) or saline (control oedema) was orally administered in two different times: 30 min before (a) and 1 h after (b) i.pl. injection of cg. The increase paw size was measured 1, 2, 3, 4, and 5 h after cg injection. The time zero corresponds to cg injection. The results were expressed as mean ± EPM of 8 mice. Statistically significant difference regarding saline (control group) and C3G and AF and Indomethacin groups is expressed as *P < 0.05.

The inflammatory response to subplantar oedema induced by cg in mice was significantly reduced by prior and after oral administration of AF and C3G. Figures 2(a) and 2(b) show the time course of the paw oedema after i.pl. injection of cg (0.5% m/v). Carrageenan caused progressive increase in the paw oedema 1 h after the injection, presenting the maximum peak at 4 h, decreasing to basal level after 5 h. Before and after treatment of animals with indomethacin significantly reduced cg-induced paw oedema as expected, in comparison with the respective controls (saline). C3G (4 mg/100 g body weight), administered by gavage either 30 min before or 1 h after the cg stimulus significantly decreased (*P* < 0.05) the paw oedema (around 40% and up to 80%, resp.) at the fourth hour after cg injection when compared with the control group (Figures 2(a) and 2(b)). Also, the oral administration of AF decreased the paw oedema approximately 40% in both administration times. 

The dose of AF and C3G used in the present study is ten-times lower than that necessary of the anthocyanin mix from tart cherry to suppress the 25% complete Freund's adjuvant and cg-induced paw oedema [13] but closer than ginkgo biloba extract concentration necessary to inhibit the paw oedema induced by cg in rats [34]. This fact suggested that C3G is one of the anthocyanins that presented high anti-inflammatory activity. 

It has been established that the paw oedema induced by the subplantar injection of cg is biphasic; the early phase involves the release of the mediators serotonin, histamine, and kinins, while the late phase is characterized by the infiltration of leukocytes and mediated only by prostaglandins [35]. These results suggest that the inhibitory effect of AF or C3G on oedema formation is due to the inhibition of the synthesis and/or release of these mediators, in the early phase of inflammatory effect of cg, especially by inhibiting probably cyclooxygenase products. To support this observation, the data indicate that C3G promoted similar effectiveness in suppressing oedema, when compared to the inhibitory profile of indomethacin, a COX activity inhibitor, on cg-induced inflammation. 

### 3.2. Effect of C3G and AF on Carrageenan-Induced Cellular Influx into Peritoneal Cavity

Intraperitoneal administration of cg produces a sustained increase in postcapillary *venule* permeability, thereby leading to increased cellular infiltration, particularly of neutrophils [36]. The recruitment of leukocytes from the circulation to sites of inflammation is enhanced by a series of proinflammatory mediators, such as IL-8 and vasoactive amines, ICAM and VCAM, that are produced and released into the tissue by mast cells, macrophages, and activated endothelial cells, as well as transmigrated leukocytes [36].

**Figure 3:**
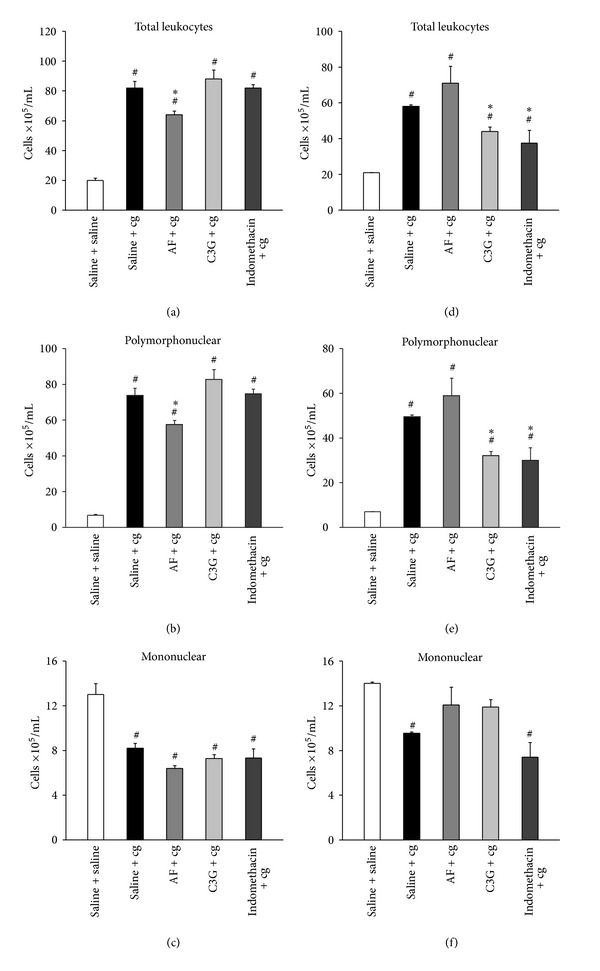
Effect of C3G and AF on carrageenan-induced leukocyte influx into peritoneal cavity. Groups of mice received C3G or AF (4 mg/100 g body weight) or indomethacin (4 mg/100 g body weight) or saline (control) by gavage in two different times: 30 min before (a, b, and c) and 1 h after (d, e, and f) cg or saline injection into the peritoneal cavity. Total leukocyte (a, d), PMN (b, e) and MN (c, f) cell counts were determined in peritoneal washes collected 3 h after cg or saline i.p. injection, as described in Section 2. Values are mean ± EPM of 8 animals. ^#^ *P* < 0.05 when compared with the corresponding group without cg stimulus (saline + saline). *P < 0.05 when compared with the corresponding control group (saline + cg).

Figure 3 presents the total leukocyte influx and differential cell into the peritoneal cavities after oral administration of C3G or AF (4 mg/100 g body weight) or indomethacin (4 mg/100 g body weight) or saline (control) 30 min before and 1 h after i.p. injection of cg (0.3% w/v) or saline solution (without stimulus). 

The oral administration of AF 30 min before the i.p. injection of cg caused a significant decrease (*P* < 0.05) in the number of total leukocytes (29% decrease) (Figure 3(a)), but not when administered 1 h after the stimulus. No reduction of total leukocytes in peritoneal exudate was observed when indomethacin was injected 30 min before cg. On the other hand, the C3G decreases the number of total leukocytes when administered 1 h after the cg stimulus (38% decrease) (Figure 3(d)). Similar effects were obtained with indomethacin administration, which promoted reduction of leukocytes (55% decrease) when administered 1 h after i.p. injection of cg. 

Differential cell counts showed that leukocytes present in the peritoneal cavity, after i.p. injection of cg, were predominantly polymorphonuclears (PMN), mainly neutrophils, when compared with the group that received saline (without stimulus). The mean values of PMN were 74 ± 4 × 10^5^ cells/mL, and 51 ± 1 × 10^5^ cells/mL, in the groups that received saline by gavage 30 min before and 1 h after cg injection, respectively (Figures 3(b) and 3(e)). On the other hand, in the group that received saline instead of cg (without stimulus), in both administration times, the mononuclear leukocytes (MN) were predominant (13 ± 1 × 10^5^ cells/mL). In addition, our results showed that cg injection caused a decrease in the number of MN in the peritoneal cavity (7.1 ± 0.1 × 10^5^ cells/mL) (Figures 3(c) and 3(f)).

Like what occurred with the total leukocytes, the number of PMN in peritoneal fluid in mice was significantly reduced when treated with C3G (39% decrease) or indomethacin (40% decrease) 1 h after the i.p cg stimulus, when compared to the control group that received saline orally (Figure 3(e)). On the other hand, AF administered 30 min before cg, promoted a significant decrease in the recruited PMN (24% decrease), compared to the control group (Figure 3(b)).

These results were different from those observed in other tissues, such as air pounch cg inflammation in mice and acute lung inflammation in rats where a decrease in the influx of cell was observed when C3G was previously administered before the cg stimulus [14, 17].

In relation to MN influx, C3G or AF or indomethacin administrated 30 min before cg injection did not change the decrease counts of MN promoted by cg injection (Figures 3(c) and 3(f)), when compared with the group without cg stimulus. 

Since C3G was detected intact and in low concentration in plasma of rats after mulberry juice intake [20], the oral intake performed 1 h after cg stimulus probably could provide an ideal concentration of C3G in plasma, resulting in the observed effect. However, this experimental protocol showed that AF is more effective than C3G as a preventive compound against leukocyte migration, suggesting that the complex mixtures of anthocyanins in AF may provide antileukocyte influx effect mainly through a combination of additive and/or synergistic effects. 

### 3.3. Effect of C3G and AF on Carrageenan-Induced Cyclooxygenase-2 Expression in Peritonitis

**Figure 4:**
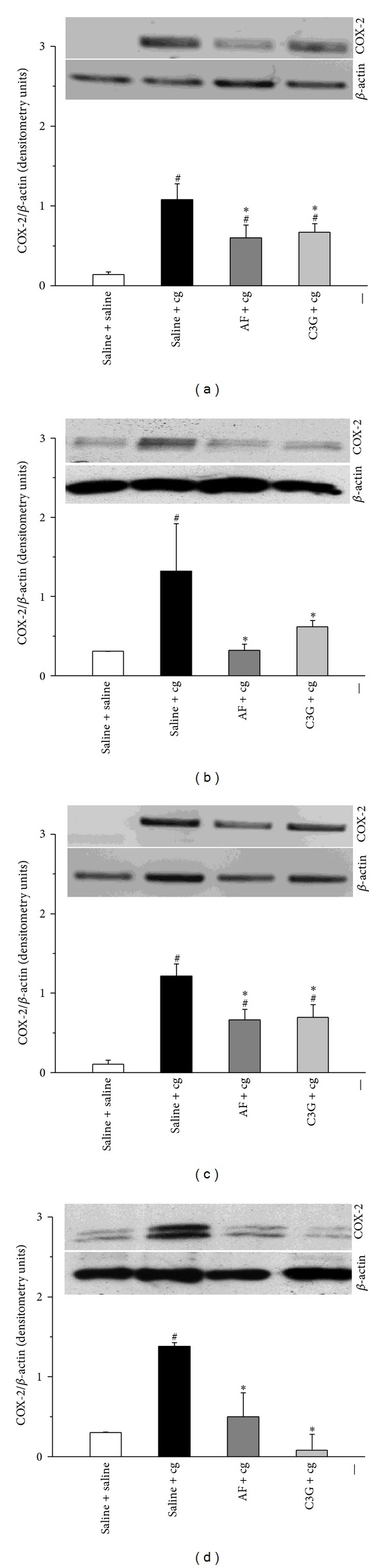
Effect of C3G and AF on carrageenan-induced cyclooxygenase-2 expression in peritoneal leukocytes. Groups of mice received C3G or AF (4 mg/100 g body weight) or saline by gavage in two different times: 30 min before or 1 h after cg (0.3% w/v) or saline injection into the peritoneal cavity. Peritoneal leukocytes were collected 3 h after i.p. administration of either cg or saline and whole cells were analyzed for COX-2 expression by RT-PCR and western blotting performed, as described in Section 2. (a and c) RT-PCR of COX-2, and *β*-actin (loading control); Bar graph shows densitometric analysis of mRNA COX-2. (b and d) Western blotting of COX-2, and *β*-actin (loading control) of leukocytes present in the inflammatory exudates; bar graph shows densitometric analysis of protein COX-2. The densities (in densitometry units) were normalized with those of *β*-actin. Results were expressed as mean ± EPM from 8 mice. ^#^ *P* < 0.05 when compared with the corresponding group without cg stimulus (saline + saline). *P < 0.05 when compared with the corresponding control group (saline + cg).

The effect of C3G or AF (4 mg/100 g body weight) on cg-induced COX-2 transcription was measured in peritoneal leukocytes by RT-PCR. As shown in Figures 4(a) and 4(c), the i.p. injection of cg (0.3% w/v) drastically increased COX-2 mRNA and protein expression. On the other hand, the oral administration of C3G and AF, either 30 min before and 1 h after cg i.p. injection, clearly downregulated COX-2 mRNA expression (50% reduction) and decreased the levels of COX-2 protein expression, when compared with the control group. 

Although some studies have documented that anthocyanins inhibit COX-2 expression in human keratinocyte cell line [15] and cultured macrophages [37, 38] and in asthma model [16], our study provides the first evidence that an anthocyanin mixture or C3G can inhibit, both preventively and therapeutically, the expression of COX-2 protein with a single oral dose. Several lines of evidence clearly established, in *in vitro* models, that the inhibition of some inflammatory cytokines [12, 16] and inhibition of activation of nuclear factor pathway, such as NF-*κ*B [10, 15], could explain the mechanisms of action of anthocyanins on the inhibition of COX-2 expression. 

Also, some sources of anthocyanins, such as black soybean anthocyanin and anthocyanins from sweet purple have showed inhibition the COX-2 expression through NF-*κ*B inhibition when administered before the stimulus in inflammation models [11, 12].

### 3.4. Effect of C3G and AF on Carrageenan-Released PGE_2_ in Peritonitis

**Figure 5:**
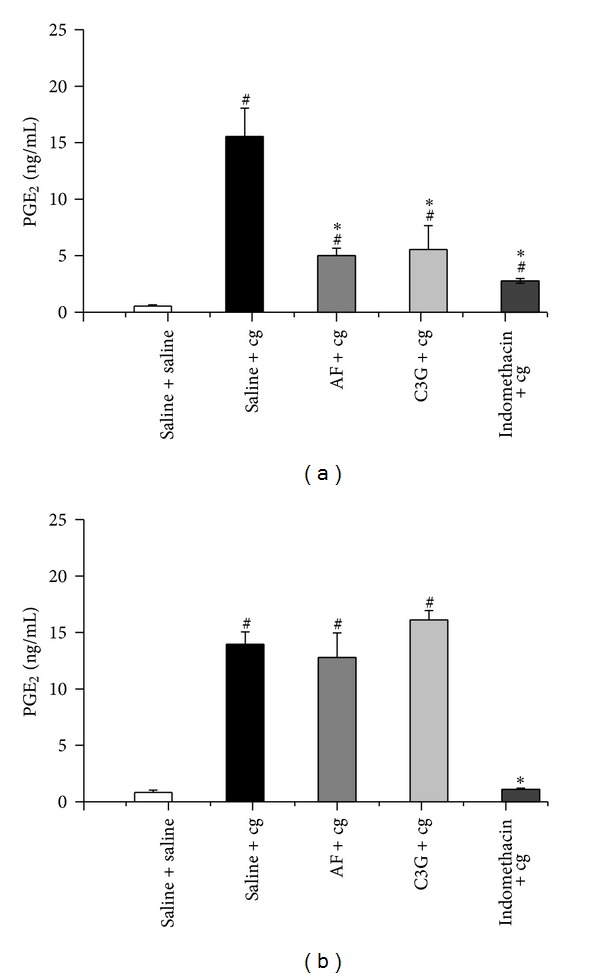
Effect of C3G and AF on carrageenan-released PGE_2_ in peritonitis. Groups of mice received C3G or AF (4 mg/100 g body weight) or indomethacin (4 mg/100 g body weight) or saline (control) by gavage in two different times: 30 min before (a) or 1 h after (b) cg or saline (control) injection into the peritoneal cavity. PGE_2_ was quantified in peritoneal exudates collected after 3 h of cg or saline administration. Values are mean ± EPM of 8 mice. ^#^ *P* < 0.05 when compared with the corresponding group without cg stimulus (saline + saline). *P < 0.05 when compared with the corresponding control group (saline + cg).

Further, this study investigated the effect of C3G and AF (4 mg/100 g body weight) on PGE_2_ production, the main inflammatory prostaglandin produced by COX activity, in peritoneal exudates from mice induced by cg. Figures 5(a) and 5(b) showed that i.p. administration of cg induced more than a 25-fold (14.5 ± 2.5 ng/mL) increase in PGE_2_ generation compared with the groups without the cg stimulus (0.50 ± 0.05 ng/mL). The PGE_2_ concentration was significantly decreased by the oral pretreatment with C3G, AF, and indomethacin, 30 min before cg injection (4.5 ± 1.0 ng/mL, 5.0 ± 2.0 ng/mL and 2.1 ± 0.1 ng/mL, resp.). In this administration time, the AF and C3G promoted approximately 70% reduction in PGE2 production by cg (Figure 5(a)). On the other hand, the oral treatment of AF or C3G, 1 h after i.p. injection of cg, did not induce any modification in the high levels of PGE_2_ release by cg (Figure 5(b)). However, in such experimental condition, the indomethacin suppressed the PGE_2_ production by cg stimulus. 

Prostaglandin E_2_ is a product generated by cyclooxygenases from arachidonic acid, and it is an important mediator in the inflammatory process. In this study, it was observed that after 3 h of administration, cg produced an increase in PGE_2_ levels into peritoneal cavity. In parallel, the results showed that C3G produced significant inhibition of PGE_2_ production when injected 30 min before cg. However, C3G did not produce such equivalent effectiveness towards cg-induced PGE_2_ release when administered 1 h after cg injection. These results are curious because in both administration times used in the present study it was possible to observe that the oral intake of C3G was effective in inhibiting COX-2 expression. Therefore, this suggests that although COX-2 mRNA and protein expression were detected at 3 h after cg injection, this isoform of COX did not present catalytic activity in this period of time. In fact, studies have demonstrated that cg-induced PGE_2_ are produced by COX-1 in the first phase, while COX-2-derived PGE_2_ turned to be involved in the second phase induced by cg injection [35]. In parallel, our data demonstrated that indomethacin was effective to inhibit PGE_2_ production in both administration times. Although it is generally accepted that nonsteroidal anti-inflammatory drugs such as aspirin and indomethacin are inhibitors of activity of both isoforms of COXs, it is known that these compounds inhibit COX-1 activity more potently than COX-2 in broken cells and in intact cells of mice [39, 40]. In addition, the absence of PGE_2_ inhibition when C3G was administered 1 h after cg stimulus compared to the preventive effect obtained by C3G when administered 30 min before the stimulus may be a reflection of the plasma concentrations of this anthocyanin in each administration time. 

## 4. Conclusions

In the present study, AF and C3G have been found to be prophylactic or therapeutically efficient on suppressing cg-induced acute inflammation in mice, like oedema and peritonitis, demonstrating to be an anti-inflammatory component from *Morus nigra*. The results suggest that the anti-inflammatory properties of AF and its major component, the C3G, might be correlated to inhibition of the PMN influx, to downregulation of COX-2 expression, and to inhibition of PGE_2_ production.
